# Comprehensive In Vitro Metabolic Characterization of Eudesmin in Human and Mouse Hepatocytes

**DOI:** 10.3390/pharmaceutics18040432

**Published:** 2026-03-31

**Authors:** Min Seo Lee, Ju-Hyun Kim, Im-Sook Song, Yong-Yeon Cho, Joo Young Lee, Hye Suk Lee

**Affiliations:** 1 College of Pharmacy, The Catholic University of Korea, Bucheon 14662, Republic of Korea; minseo.lee@catholic.ac.kr (M.S.L.); yongyeon@catholic.ac.kr (Y.-Y.C.); joolee@catholic.ac.kr (J.Y.L.); 2 College of Pharmacy, Yeungnam University, Gyeongsan 38541, Republic of Korea; jhkim@yu.ac.kr; 3 BK21 FOUR Community-Based Intelligent Novel Drug Discovery Education Unit, Vessel-Organ Interaction Research Center (VOICE), Research Institute of Pharmaceutical Sciences, College of Pharmacy, Kyungpook National University, Daegu 41566, Republic of Korea; isssong@knu.ac.kr

**Keywords:** eudesmin, metabolite identification, hepatocytes, drug-metabolizing enzymes, ion identity molecular networking

## Abstract

**Background/Objectives:** Eudesmin is a tetrahydrofurofuranoid lignan known for its diverse pharmacological activities, including anti-tumor, anti-inflammatory, and neuroprotective effects. However, its metabolism has not been well characterized. **Methods:** This study examined the in vitro metabolism of eudesmin using human and mouse hepatocytes, human liver microsomes, and recombinant drug-metabolizing enzymes. Liquid chromatography–high-resolution mass spectrometry combined with ion identity molecular networking enabled the comprehensive visualization and annotation of eudesmin metabolites. **Results:** Eudesmin exhibited moderate metabolic stability in human and mouse hepatocytes, with half-lives of 181.0 min and 132.9 min, and intrinsic clearance values of 27.7 mL/min/kg and 154.0 mL/min/kg, respectively. Incubation of eudesmin with human hepatocytes resulted in the formation of 13 metabolites, including five phase I metabolites (M1–M5) and eight phase II conjugates. Phase I metabolism was dominated by *O*-demethylation of the 3,4-dimethoxyphenyl moieties, yielding mono-*O*-demethylated (M1 and M2) and di-*O*-demethylated metabolites (M3 and M4), as well as a hydroxylated metabolite (M5). Enzyme phenotyping, kinetic analyses, and chemical inhibition experiments identified cytochrome P450 2C9 (CYP2C9) as the major contributor to *O*-demethylation, with additional contributions from CYP2C19, CYP2C8, CYP3A4, and CYP3A5, whereas hydroxylation was mediated primarily by CYP3A4 and CYP3A5. The *O*-demethylated metabolites subsequently underwent phase II metabolism, forming glucuronide conjugates of M1–M4 and sulfate conjugates of M1–M3, including a disulfate of M3. Uridine 5′-diphospho-glucuronosyltransferase and sulfotransferase screening revealed the involvement of multiple conjugative enzymes, indicating extensive and distributed phase II metabolism. Specifically, di-*O*-demethylated metabolites and their conjugates were detected in human hepatocytes but not in mouse hepatocytes, suggesting that the sequential *O*-demethylation pathway is limited in mice. **Conclusions:** This study characterizes eudesmin metabolism, with CYP2C9-mediated *O*-demethylation and significant species differences between humans and mice, and provides a basis for its further pharmaceutical development.

## 1. Introduction

Eudesmin is a tetrahydrofurofuranoid lignan predominantly found in *Magnoliae flos* and *Zanthoxylum armatum* [1,2]. Extensive in vitro and in vivo studies have revealed its pharmacological properties, including the induction of apoptosis in lung cancer cells via Akt/JNK signaling and the reversal of P-glycoprotein-mediated multidrug resistance [3,4,5]. In addition, eudesmin has been reported to exhibit gastroprotective, anti-inflammatory, and neuroprotective activities [6,7]. Neurologically, it exerts anticonvulsant and sedative activities via GABAergic modulation while also promoting vascular relaxation through endothelial histamine H1 receptor activation [8,9]. Furthermore, eudesmin inhibits adipogenic differentiation by suppressing the S6K1 signaling pathway [10]. Despite the therapeutic potentials, the lack of information on systemic biotransformation remains an unresolved aspect in the evaluation of eudesmin as a drug candidate. Characterizing the metabolic fate of a compound is essential for predicting its pharmacokinetic behavior, efficacy, and safety profile in humans [11].

Current literature on the drug–drug interaction (DDI) potential of eudesmin is limited primarily to its role as a perpetrator. Previous studies have identified the inhibitory effects of eudesmin on uridine 5′-diphospho-glucuronosyltransferase 1A1 (UGT1A1)- and UGT1A3-mediated glucuronidation, with *K*_i_ values of 25.7 µM and 39.8 µM, respectively, while showing negligible inhibition toward other major UGT isoforms (UGT1A4, UGT1A6, UGT1A9, and UGT2B7) and eight major cytochrome P450 (CYP) enzymes [12,13]. Nevertheless, the metabolic clearance of eudesmin as a substrate or victim of DDIs has not been established. Considering the structural similarity of eudesmin to other tetrahydrofurofuranoid lignans that undergo extensive first-pass metabolism [14,15,16,17,18], identifying its biotransformation pathways and the enzymes responsible for its clearance is a critical requirement for understanding its disposition.

The structural complexity of the tetrahydrofurofuranoid lignans, characterized by various methoxy substitution patterns, presents challenges for conventional metabolite identification. Traditional targeted approaches often prove insufficient for the comprehensive identification of minor or unexpected metabolites [19]. Previous research on structurally related kobusin reported that the integration of high-resolution mass spectrometry (HRMS) with molecular network-based analytical frameworks is necessary for the precise elucidation of metabolic pathways [18]. Leveraging this analytical framework, the present study used ion identity molecular networking (IIMN) combined with liquid chromatography–high-resolution mass spectrometry (LC–HRMS). This approach enables the unbiased visualization and systematic annotation of the metabolic network, facilitating the identification of sequential phase I and phase II metabolites.

This study characterized the in vitro metabolism of eudesmin using human and mouse hepatocytes, human liver microsomes (HLMs), and recombinant enzymes. Thirteen metabolites were identified using an IIMN-integrated approach, including the bioactive metabolite pinoresinol, and the specific CYP, UGT, and sulfotransferase (SULT) isoforms involved in their formation were determined. Furthermore, this study evaluated the interspecies metabolic differences between humans and mice. These results provide the first comprehensive metabolic framework for eudesmin, offering the necessary data for its future pharmacological evaluation and clinical application.

## 2. Materials and Methods

### 2.1. Materials

Eudesmin (purity, 99.4%) and epimagnolin A (purity, 98.1%, internal standard) were obtained from MedChemExpress (Monmouth Junction, NJ, USA). Pinoresinol (purity, 98.0%) was purchased from Tokyo Chemical Industry (Tokyo, Japan), and *O*-methylpinoresinol (purity, 92.0%) was acquired from TargetMol (Boston, MA, USA). Alamethicin, nicotinamide adenine dinucleotide phosphate, glucose-6-phosphate, glucose-6-phosphate dehydrogenase, 3-phosphoadenosine-5-phosphosulfate (PAPS), uridine 5′-diphosphoglucuronic acid (UDPGA), potassium phosphate dibasic trihydrate, potassium phosphate monobasic, tris base, tris hydrochloride, magnesium chloride (MgCl_2_), β-glucuronidase from *Escherichia coli*, α-naphthoflavone, tranylcypromine, thiotepa, sulfaphenazole, omeprazole, quinidine, diethyldithiocarbamate, and ketoconazole were obtained from Sigma-Aldrich Co. (St. Louis, MO, USA). Montelukast was obtained from Supelco (Bellefonte, PA, USA). Pooled HLM, pooled human liver S9 fraction, human cDNA-expressed CYP enzymes (CYP1A2, CYP2A6, CYP2B6, CYP2C8, CYP2C9, CYP2C19, CYP2D6, CYP2E1, CYP3A4, and CYP3A5), and human cDNA-expressed UGT enzymes (UGT1A1, UGT1A3, UGT1A4, UGT1A6, UGT1A7, UGT1A8, UGT1A9, UGT1A10, UGT2B4, UGT2B7, UGT2B10, UGT2B15, and UGT2B17) were obtained from Corning Life Sciences (Woburn, MA, USA). Cryopreserved human and mouse hepatocytes, opti-incubate hepatocyte media, and OptiThaw cryohepatocyte kit were purchased from XenoTech (Kansas City, KS, USA). Human cDNA-expressed SULT enzymes (SULT1A1*1, SULT1A1*2, SULT1A2, SULT1A3, SULT1B1, SULT1C2, SULT1C4, SULT1E1, and SULT2A1) were supplied by Cypex (Dundee, Scotland, UK). Acetonitrile, methanol, and water (LC-MS grade) were purchased from Fischer Scientific (Fair Lawn, NJ, USA). All other chemicals were of the highest quality available.

### 2.2. Metabolic Stability of Eudesmin in Human and Mouse Hepatocytes

The metabolic stability of eudesmin was investigated in human and mouse hepatocytes following the method of Lee et al. [16,17]. Briefly, 60 µL of hepatocyte suspensions (final concentration: 1.0 × 10^6^ cells/mL) and 60 µL of eudesmin (2 µM) prepared in incubation medium were mixed in 96-well plates. The mixtures were incubated in triplicate at 37 °C for 10, 20, 30, 60, 90, and 120 min in a CO_2_ incubator. The reactions were quenched by adding 120 µL of ice-cold acetonitrile containing epimagnolin A (internal standard, 100 ng/mL). The samples were sonicated for 10 min and centrifuged at 13,000 rpm for 10 min at 4 °C. Subsequently, 5 µL of the supernatant was injected into the LC–MS/MS system. Honokiol was used as a positive control to validate the hepatocyte incubation system [20].

### 2.3. Characterization of Eudesmin Metabolism in Hepatocytes and Liver S9 Fractions

To identify eudesmin metabolites, 60 µL aliquots of human or mouse hepatocyte suspensions (1.0 × 10^6^ cells/mL) were mixed with an equal volume of eudesmin (20 µM) prepared in incubation medium in 96-well plates and incubated at 37 °C for 2 h in a CO_2_ incubator. Control samples were quenched immediately after mixing (0 h).

To definitively characterize the phase II metabolic pathways identified in the hepatocytes, biotransformation assays were further conducted using pinoresinol, a key intermediate metabolite. Briefly, pinoresinol (10 µM) was incubated with human liver S9 fractions (0.5 mg/mL) in potassium phosphate buffer (50 mM, pH 7.4) containing MgCl_2_ (10 mM). To specifically target glucuronidation and sulfation pathways, the reaction mixtures were supplemented with either UDPGA (1 mM) with alamethicin (25 µg/mg protein) or PAPS (0.4 mM), respectively, and incubated at 37 °C for 1 h.

For enzymatic hydrolysis of glucuronide conjugates, dried human hepatocyte samples were reconstituted in 50 mM potassium phosphate buffer (pH 6.8) containing β-glucuronidase (200 units/mL) and incubated at 37 °C for 1 h. The control samples were incubated under identical conditions in the absence of β-glucuronidase.

All reactions were terminated by adding ice-cold acetonitrile. The mixtures were sonicated for 10 min and centrifuged at 13,000 rpm for 10 min at 4 °C. The supernatants were evaporated to dryness under a gentle stream of nitrogen and reconstituted in 100 µL of 30% methanol. Aliquots (5 µL) were injected into the LC–HRMS system for analysis.

### 2.4. Ion Identity Molecular Networking

For IIMN analysis, raw mass spectrometry files were converted to mzML format using MSConvert (ProteoWizard) and subsequently processed in MZmine (version 4.8.5) via the MZWizard workflow, which enables automated chromatographic feature detection, alignment, and preprocessing for IIMN applications [21,22]. The chromatographic processing parameters included a maximum of 15 peaks per chromatogram, a minimum of five consecutive scans, an estimated feature FWHM of 0.2 min, and retention time tolerances of 0.04 min for intra-sample alignment and 0.1 min for inter-sample alignment. For Orbitrap data processing, noise thresholds were set to 5 × 10^4^ for MS^1^ and 1 × 10^3^ for MS^2^, with a minimum feature height of 1.5 × 10^5^. The *m*/*z* tolerances were defined as 5, 3, and 5 ppm for scan-to-scan detection, intra-sample alignment, and inter-sample alignment, respectively. Ion identity networking was performed using an *m*/*z* tolerance of 3 ppm, allowing a maximum charge state of 2 and up to two molecules per cluster. The considered adducts included [M+H]^+^, [M+NH_4_]^+^, [M+Na]^+^, [M+K]^+^, [M+H-H_2_O]^+^, and [M+H-2H_2_O]^+^. Aligned feature intensity tables (.csv) and corresponding MS/MS spectra (.mgf) were exported and uploaded to the Global Natural Products Social Molecular Networking 2 (GNPS2) platform (https://gnps2.org; accessed on 5 February 2026) for IIMN analysis [23]. Molecular networks were generated using a precursor and fragment ion mass tolerance of 0.02 Da, a minimum of four matched fragment ions, and a cosine similarity threshold greater than 0.6. Network visualization was conducted using Cytoscape (version 3.9.1) [24].

### 2.5. Characterization of CYP Enzymes Involved in Eudesmin Metabolism

For screening the CYP enzymes involved in the phase I metabolism of eudesmin, incubation mixtures (95 μL) containing eudesmin (final concentration, 5 μM), 10 mM MgCl_2_, and individual human cDNA-expressed CYP enzymes (CYP1A2, CYP2A6, CYP2B6, CYP2C8, CYP2C9, CYP2C19, CYP2D6, CYP2E1, CYP3A4, and CYP3A5; 4 pmol) in 50 mM potassium phosphate buffer (pH 7.4) were incubated at 37 °C for 30 min in a shaking water bath after adding an NADPH generating system.

To evaluate enzyme kinetic parameters, incubation mixtures (95 μL) containing various concentrations of eudesmin (final concentration, 2, 5, 10, 25, 50, 100, 150, 200, 350, or 500 μM), 10 mM MgCl_2_, pooled HLM (final concentration, 0.2 mg/mL) or individual human cDNA-expressed CYP2C8, CYP2C9, CYP2C19, CYP3A4, or CYP3A5 enzymes (2 pmol) in 50 mM potassium phosphate buffer (pH 7.4) were preincubated for 3 min at 37 °C. The reactions were initiated by adding an NADPH-generating system and further incubated at 37 °C for 20 min in a shaking water bath.

Chemical inhibition experiments were performed by incubating pooled HLM (0.2 mg/mL) with eudesmin (10 μM), 10 mM MgCl_2_, an NADPH-generating system, and selective CYP inhibitors in 100 mM potassium phosphate buffer (pH 7.4) at 37 °C for 30 min in a shaking water bath. The inhibitors used were α-naphthoflavone (1 μM, CYP1A2), tranylcypromine (1 μM, CYP2A6), thiotepa (10 μM, CYP2B6), montelukast (1 μM, CYP2C8), sulfaphenazole (10 μM, CYP2C9), omeprazole (10 μM, CYP2C19), quinidine (1 μM, CYP2D6), diethyldithiocarbamate (50 μM, CYP2E1), and ketoconazole (0.5 μM, CYP3A4).

All reactions were terminated by adding 100 μL of epimagnolin A (internal standard, 100 ng/mL) in methanol. The samples were centrifuged at 13,000 rpm for 10 min at 4 °C. Aliquots (5 μL) of the resulting supernatants were injected into the LC–MS/MS system.

### 2.6. Screening of UGT and SULT Enzymes Involved in the Glucuronidation of M3

For UGT screening, aliquots (95 μL) of Tris buffer (50 mM, pH 7.4) containing human cDNA-expressed UGT enzymes (UGT1A1, UGT1A3, UGT1A4, UGT1A6, UGT1A7, UGT1A8, UGT1A9, UGT1A10, UGT2B4, UGT2B7, UGT2B10, UGT2B15, and UGT2B17; 10 μg protein each) and alamethicin (2.5 μg/mL) were incubated with pinoresinol (M3, 5 μM). The reactions were initiated by adding UDPGA (5 μL, 5 mM) and incubated at 37 °C for 30 min in a shaking water bath.

For SULT screening, aliquots (95 μL) of potassium phosphate buffer (50 mM, pH 7.4) containing human cDNA-expressed SULT enzymes (SULT1A1*1, SULT1A1*2, SULT1A2, SULT1A3, SULT1B1, SULT1C2, SULT1Cg SULT1E1, and SULT2A1; 0.5 μg protein each) were incubated with pinoresinol (M3, 5 μM). The reactions were initiated by adding PAPS (5 μL, 0.4 mM) and incubated at 37 °C for 30 min in a shaking water bath.

All reactions were terminated by adding 100 μL of epimagnolin A (internal standard, 100 ng/mL in methanol). The samples were centrifuged at 13,000 rpm for 10 min at 4 °C. Aliquots (5 μL) of the resulting supernatants were injected into the LC–MS/MS system.

### 2.7. Analysis of Eudesmin and Its Metabolites via LC-MS/MS

An Agilent 6495 Triple Quadrupole MS coupled to a 1290 Infinity LC system (Agilent Technologies, Wilmington, DE, USA) was used to quantify eudesmin and its metabolites. Chromatographic separation was achieved on a Halo C18 column (2.1 × 100 mm, 2.7 μm; Advanced Material Technology, Wilmington, DE, USA) using mobile phase A (5% methanol in 0.1% formic acid) and mobile phase B (95% methanol in 0.1% formic acid). Different LC gradient programs and flow rates were applied depending on the experimental purpose. For the metabolic stability and CYP-related experiments, the separation was performed at a flow rate of 0.25 mL/min with the following gradient: 45% B for 1 min, 45–60% B over 5 min, 60–90% B over 1 min, 90% B for 3 min, 90–45% B over 0.1 min, and 45% B for 2.9 min. For SULT screening experiments, the flow rate was set to 0.30 mL/min, and the gradient program consisted of 10% B for 0.5 min, 10–90% B over 6.5 min, 90% B for 3 min, 90–10% B over 0.1 min, and 10% B for 2.9 min. For UGT screening experiments, the separation was also performed at a flow rate of 0.30 mL/min using the following gradient: 30% B for 0.5 min, 30–72% B over 6.8 min, 72–90% B over 0.1 min, 90% B for 3 min, 90–30% B over 0.1 min, and 30% B for 2.9 min. The autosampler and column temperatures were 4 °C and 40 °C, respectively. Electrospray ionization in positive ion mode was used, and the electrospray source settings were as follows: gas temperature, 250 °C; gas flow, 14 L/min; nebulizer, 30 psi; sheath gas temperature, 300 °C; sheath gas flow, 12 L/min; capillary voltage, 3500 V; and nozzle voltage, 1000 V. The multiple reaction monitoring transitions were *m*/*z* 369.2 → 298.0 for eudesmin, *m*/*z* 355.1 → 136.8 for M1 and M2, *m*/*z* 341.0 → 271.1 for M3 and M4, *m*/*z* 403.0 → 151.1 for M5, *m*/*z* 552.2 → 235.0 for M3-G, *m*/*z* 420.9 → 137.1 for M3-S1, *m*/*z* 535.9 → 314.9 for M3-S2, and *m*/*z* 399.2 → 328.1 for epimagnolin A (internal standard) at collision energies of 20, 20, 15, 15, 15, 16, 10, and 25 eV, respectively. Eudesmin, M1, and M3 were quantified using authentic standards. M2 and M5 were quantified using the calibration curve of M1, whereas M4, M3-G, M3-S1, and M3-S2 were quantified using the calibration curve of M3.

### 2.8. Identification of Eudesmin Metabolites via LC-HRMS

A Q-Exactive Orbitrap mass spectrometer (Thermo Scientific, Waltham, MA, USA) coupled to a Nexera X2 UPLC (Shimadzu, Kyoto, Japan) was used for the structural identification of eudesmin and its potential metabolites. The analytes were separated on a Halo C18 column (2.1 × 100 mm, 2.7 μm; Advanced Material Technology, Wilmington, DE, USA) using a gradient elution with mobile phase A (5% methanol in 10 mM ammonium formate) and mobile phase B (95% methanol) at a flow rate of 0.3 mL/min. The gradient program was as follows: 22% B for 7 min, 22–28% B over 7 min, 28–44% B over 8 min, 44–90% B over 8 min, 90% B for 3 min, 90–22% B over 0.1 min, and 22% B for 3.9 min. The autosampler and column temperatures were 4 °C and 40 °C, respectively. Positive electrospray ionization was used, and the electrospray source settings were as follows: aux gas heater temperature, 300 °C; capillary temperature, 350 °C; spray voltage, 3.5 kV; nitrogen sheath gas, 40 arbitrary units; auxiliary gas, 10 arbitrary units; and collision dissociation energy, 10 eV. Full-scan MS1 with data-dependent MS2 acquisition mode was used to obtain MS scan data, ranging from *m*/*z* 100 to 1000 with a resolution of 70,000. The data were processed using Xcalibur software version 2.2 (Thermo Scientific, Waltham, MA, USA). The product ion structures were determined using Mass Frontier software (version 6.0; HighChem Ltd., Bratislava, Slovakia).

### 2.9. Data Analysis and Statistical Analysis

The apparent in vitro half-life (*t*_1/2_) of eudesmin was determined from the slope of the natural logarithm of the percentage of the parent compound remaining versus the incubation time, assuming first-order elimination kinetics. The elimination rate constant (*k*) was obtained as the negative value of the slope, and *t*_1/2_ was calculated using the equation *t*_1/2_ = ln2/*k*, intrinsic clearance (*CL*_int_), and hepatic clearance (*CL*_hep_) of eudesmin in human and mouse hepatocytes, calculated using the following equations:

$${CL}_{int}(mL/min/kg)=\frac{ln2}{t_{1/2}}\times \frac{mL\;incubation}{hepatocyte\;(10^{6}\;cells)}\times \frac{B\times 10^{6}\;cells}{g\;liver}\times \frac{A\;g\;liver}{kg\;BW}$$

$${CL}_{hep}(mL/min/kg)=\frac{Q_{h}\times {CL}_{int}}{Q_{h}+{CL}_{int}}$$

In these equations, A represents the liver weight per kilogram body weight (25.7 g/kg for human and 87.5 g/kg for mouse), B represents hepatocellularity (139 × 10^6^ cells/g liver for human and 135 × 10^6^ cells/g liver for mouse), and *Q*_h_ represents the hepatic blood flow (20.7 mL/min/kg for human and 90 mL/min/kg for mouse) [25,26]. The hepatic extraction ratio was calculated using *CL*_hep_/*Q*_h_, with values of ≤0.25, 0.25–0.75, and ≥0.75 classified as low, moderate, and high extraction, respectively [27].

The metabolite concentrations were quantified, and the formation rates (pmol/mg protein or pmol CYP/min) were calculated by dividing the amount of each metabolite formed in incubations with HLM or human cDNA-expressed CYP enzymes by the incubation time. For enzyme kinetic analysis, the metabolite formation rates were plotted as a function of the eudesmin concentration, and the kinetic parameters, including *K*_m_, *V*_max_, *K*_i_, and Hill coefficient, were calculated using enzyme kinetics software (SigmaPlot, version 12.5; Systat Software Inc., San Jose, CA, USA).

The relative contributions of the individual CYP isoforms to the formation of metabolites M1–M5 from eudesmin in HLM were calculated using the following equation: (Velocity_CYPi_/∑Velocity_CYPi_ × RAF_CYPi_) × 100. In this equation, the relative activity factor (RAF) accounts for differences in hepatic abundance and the catalytic activity between human cDNA-expressed CYP enzymes and HLM [28]. RAF values were calculated as the ratio of the metabolic rate of a probe substrate in HLM to that in the corresponding recombinant CYP isoforms [29]. The RAF values used in this study were 46.7, 170.4, 2.7, 18.2, and 24.7 for CYP2C8, CYP2C9, CYP2C19, CYP3A4, and CYP3A5, respectively.

## 3. Results

### 3.1. Metabolic Stability of Eudesmin in Hepatocytes

The metabolic stability of eudesmin was evaluated in human and mouse hepatocytes, and the resulting parameters are listed in Table 1. In human hepatocytes, eudesmin had a half-life (*t*_1/2_) of 181.0 min, with an intrinsic clearance (*CL*_int_) of 27.7 mL/min/kg and a hepatic clearance (*CL*_hep_) of 11.8 mL/min/kg. The hepatic extraction ratio was 0.57, indicating an intermediate level of hepatic metabolism. In mouse hepatocytes, a comparable half-life of 132.9 min was observed, along with *CL*_int_ and *CL*_hep_ values of 154.0 and 56.8 mL/min/kg, respectively. The hepatic extraction ratio in mouse hepatocytes was 0.63, indicating an intermediate extent of hepatic metabolism. Overall, eudesmin showed comparable in vitro metabolic stability in human and mouse hepatocytes, with intermediate hepatic extraction in both species. The suitability of the hepatocyte incubation system was supported by the metabolic stability of honokiol used as a positive control (Table S1).

### 3.2. IIMN-Based Metabolic Pathway Analysis

**Figure 1:**
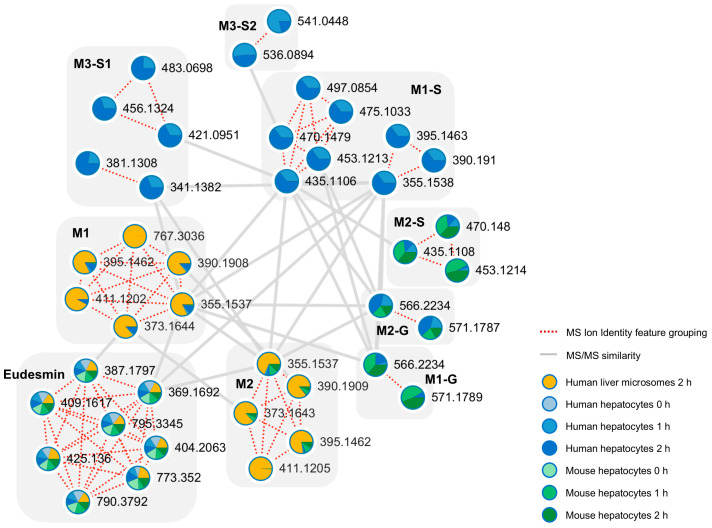
Ion identity molecular network of eudesmin metabolites generated from human liver microsomes (with NADPH) and human and mouse hepatocytes. The nodes are labeled with *m*/*z* values, and the pie charts represent the relative metabolite abundance across sample types.

The untargeted detection and organization of eudesmin-related metabolites were achieved by applying IIMN to LC–HRMS/MS datasets obtained from HLM supplemented with NADPH, human hepatocytes, and mouse hepatocytes (Figure 1). Multiple ion species originating from eudesmin were consolidated into a single ion identity family, including [M+H-H_2_O]^+^, [M+H]^+^, [M+NH_4_]^+^, [M+Na]^+^, [M+K]^+^, [2M+H]^+^, [2M+NH_4_]^+^, and [2M+Na]^+^. Based on MS/MS spectral similarity, eight metabolite nodes were connected to the eudesmin molecular family within the network. Interpretation of mass shifts relative to the parent compound suggested multiple biotransformation pathways, including demethylation and di-demethylation, as well as subsequent phase II conjugations such as glucuronidation and sulfation. Although certain unconjugated precursor metabolites were not represented as distinct nodes in the molecular network, the presence of their corresponding conjugates suggested their formation.

**Figure 2:**
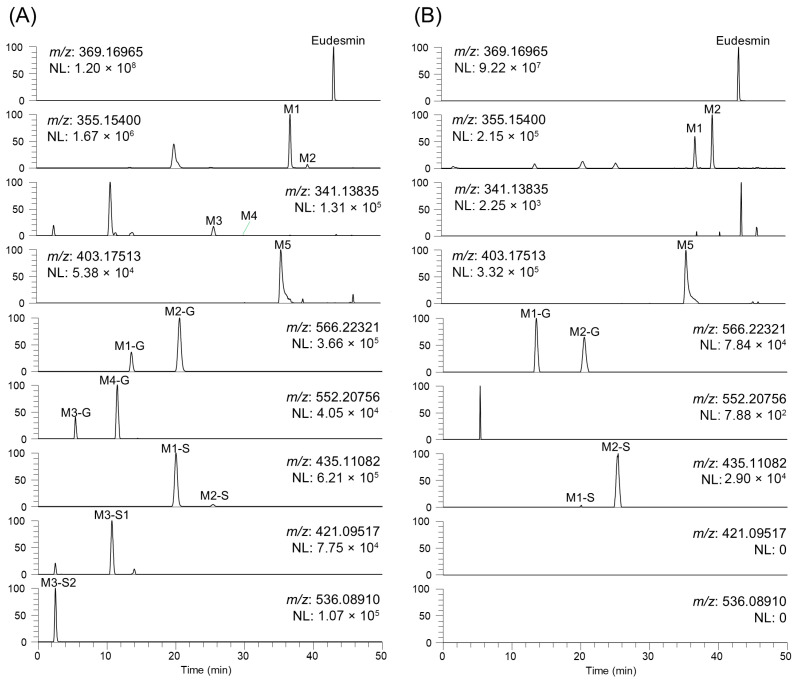
Extracted ion chromatograms of eudesmin and its metabolites after incubating 10 μM eudesmin with (**A**) human and (**B**) mouse hepatocytes at 37 °C for 2 h.

Targeted extracted ion chromatogram inspection was performed using a network-informed approach, which enabled the detection of additional metabolites that were not included in the network. The node annotations were determined using MS/MS fragmentation patterns in combination with retention time comparison to authentic standards and S9 fraction incubation experiments. Figure 2 presents the representative extracted ion chromatograms of eudesmin and possible metabolites. Table 2 lists the detailed information on the retention time (t_R_), elemental composition, precursor ions, mass errors, and product ions of eudesmin and its 13 metabolites.

**Figure 3:**
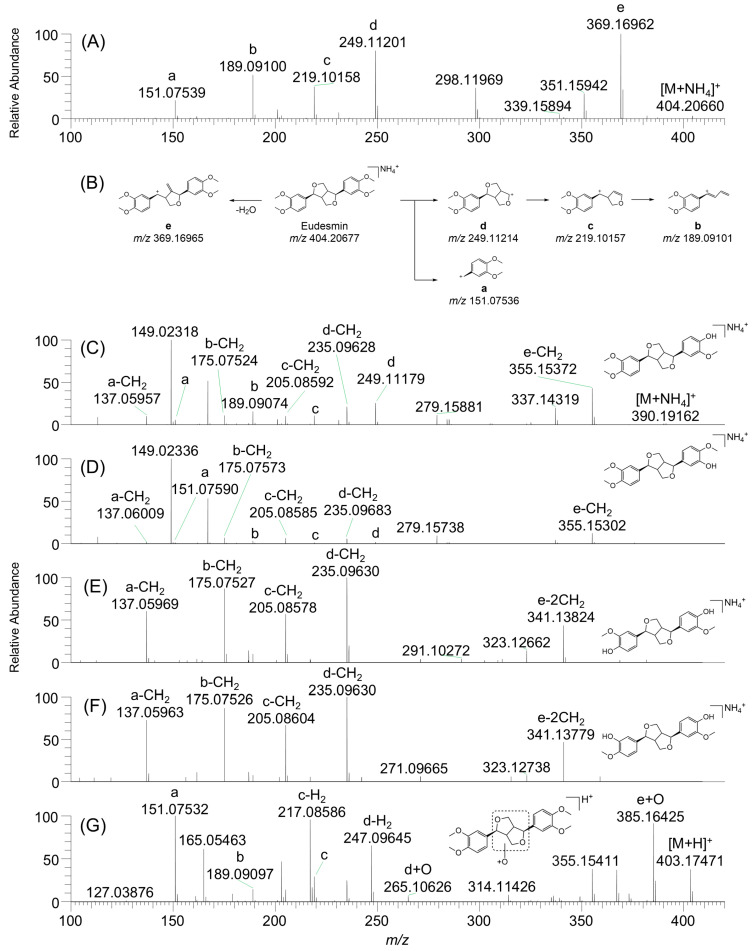
MS/MS spectra and fragmentation patterns of (**A**) eudesmin, (**C**–**G**) phase I metabolites, and (**B**) the proposed fragmentation mechanism of eudesmin. Letters a–e denote characteristic product ions of eudesmin.

The MS/MS spectrum of eudesmin ([M+NH_4_]^+^, *m*/*z* 404.20651) exhibited five characteristic product ions. The product ion at *m*/*z* 369.16962 (e) corresponded to the loss of water from the tetrahydrofuran ring. The product ions at *m*/*z* 151.07539 (a), 189.09100 (b), 219.10158 (c), and 249.11201 (d) were identified as fragment ions containing a 3,4-dimethoxyphenyl group (Figure 3A,B).

M1 and M2 ([M+NH_4_]^+^, *m*/*z* 390.19128) were identified as *O*-demethylated metabolites of eudesmin. The product ion spectra showed an unaltered 3,4-dimethoxyphenyl group, with product ions at *m*/*z* 151.07521 (a), 189.09074 (b), 219.10138 (c), and 249.11179 (d). In contrast, the ions at *m*/*z* 137.05957 (a-CH_2_), 175.07524 (b-CH_2_), 205.08592 (c-CH_2_), 235.09628 (d-CH_2_), and 355.15372 (e-CH_2_) indicated *O*-demethylation on the 3,4-dimethoxyphenyl group (Figure 3C,D). M1 was confirmed as *O*-methylpinoresinol (i.e., 4-*O*-desmethyleudesmin) based on the retention time and MS/MS spectrum being identical to those of the authentic standard. Accordingly, M2 was identified as 3-*O*-desmethyleudesmin.

M3 and M4 ([M+NH_4_]^+^, *m*/*z* 376.17499) were identified as di-*O*-demethylated metabolites of eudesmin. The product ion spectra exclusively showed ions corresponding to the guaiacol moiety, including *m*/*z* 137.05969 (a-CH_2_), 175.07527 (b-CH_2_), 205.08578 (c-CH_2_), 235.09630 (d-CH_2_), and 341.13824 (e-2CH_2_), indicating that both 3,4-dimethoxyphenyl groups had undergone *O*-demethylation (Figure 3E,F). M3 was confirmed as pinoresinol (i.e., 4′,4″-di-*O*-desmethyleudesmin) based on the retention time and MS/MS spectrum being identical to those of the authentic standard. M4 was formed from 4-*O*-desmethyleudesmin (M1) under NADPH-supplemented conditions, indicating asymmetric demethylation across the two 3,4-dimethoxyphenyl groups and supporting its assignment as 3′,4″-di-*O*-desmethyleudesmin.

M5 ([M+H]^+^, *m*/*z* 403.17538) was identified as hydroxyeudesmin. The fragment ions at *m*/*z* 217.08586 (c-H_2_), 247.09645 (d-H_2_), 265.10626 (d+O), and 385.16425 (e+O) suggest that hydroxylation occurred on the tetrahydrofuran ring, while the ions at *m*/*z* 151.07532 (a), 189.09097 (b), and 219.10172 (c) correspond to the 3,4-dimethoxyphenyl group (Figure 3G). On the other hand, the exact position of hydroxylation on the tetrahydrofuran moiety could not be determined because of the absence of an authentic standard.

M1-G and M2-G ([M+NH_4_]^+^, *m*/*z* 566.22369) were identified as *O*-desmethyleudesmin glucuronides. The product ions at *m*/*z* 137.05952 (a-CH_2_), 151.07536 (a), 175.07541 (b-CH_2_), 189.09113 (b), 205.08612 (c-CH_2_), 219.10159 (c), 235.09647 (d-CH_2_), 249.11214 (d), and 355.15408 (e-CH_2_) were consistent with the fragment ions observed for M1 and M2 (Figure S1). M1-G was confirmed as the glucuronide of M1 based on the retention time and MS/MS spectrum being identical to those of 4-*O*-desmethyleudesmin glucuronide, which was produced by incubating *O*-methylpinoresinol with human liver S9 fractions in the presence of UDPGA at 37 °C for 1 h (Figure S2A). Accordingly, M2-G was presumed to be the glucuronide of M2.

M3-G and M4-G ([M+NH_4_]^+^, *m*/*z* 552.20862) were identified as di-*O*-desmethyleudesmin glucuronides. The product ions at *m*/*z* 137.05975 (a-CH_2_), 175.07547 (b-CH_2_), 205.08609 (c-CH_2_), 235.09648 (d-CH_2_), and 341.13846 (e-2CH_2_) were consistent with the fragment ions observed for M3 and M4 (Figure S1). M3-G was confirmed to be the glucuronide of M3 based on the retention time and MS/MS spectrum being identical to those of pinoresinol glucuronide, which was produced by incubating pinoresinol with human liver S9 fractions in the presence of UDPGA (Figure S2C). M4-G was assigned as the glucuronide of M4, supported by enzymatic hydrolysis with β-glucuronidase, which resulted in a marked decrease in the M4-G signal and an increase in M4 (Figure S3). However, the exact site of glucuronidation could not be determined based on the available data.

M1-S and M2-S ([M+NH_4_]^+^, *m*/*z* 470.14838) were identified as *O*-desmethyleudesmin sulfates. The product ions at *m*/*z* 217.01657 (a-CH_2_+SO_3_), 255.03215 (b-CH_2_+SO_3_), 285.04257 (c-CH_2_+SO_3_), 315.05359 (d-CH_2_+SO_3_), and 435.11121 (e-CH_2_+SO_3_) indicated that sulfation occurred on the guaiacol moiety. The product ions at *m*/*z* 137.05954 (a-CH_2_), 151.07553 (a), 175.07542 (b-CH_2_), 235.09653 (d-CH_2_), 249.11153 (d), and 355.15402 (e-CH_2_) were consistent with the fragment ions of M1 and M2 (Figure S1). M1-S was assigned as the sulfate conjugate of M1 based on the retention time and MS/MS fragmentation matching with those of *O*-methylpinoresinol sulfate, which was generated by incubating *O*-methylpinoresinol with human liver S9 fractions in the presence of PAPS at 37 °C for 1 h (Figure S2B). Accordingly, M2-S was designated as the sulfate conjugate of M2.

M3-S1 ([M+NH_4_]^+^, *m*/*z* 456.13239) was identified as di-*O*-desmethyleudesmin sulfate. The product ions at *m*/*z* 217.01608 (a-CH_2_+SO_3_), 255.03220 (b-CH_2_+SO_3_), 285.04297 (c-CH_2_+SO_3_), 315.05316 (d-CH_2_+SO_3_), and 421.09561 (e-CH_2_+SO_3_) suggested that sulfation occurred on the guaiacol moiety. The product ions at *m*/*z* 137.05975 (a-CH_2_), 175.07561 (b-CH_2_), 205.08652 (c-CH_2_), 235.09636 (d-CH_2_), and 341.13803 (e-2CH_2_) were consistent with the fragment ions of M3 (Figure S1).

M3-S2 ([M+NH_4_]^+^, *m*/*z* 536.08972) was identified as di-*O*-desmethyleudesmin disulfate. The product ions at m/z 217.01642 (a-CH_2_+SO_3_), 255.03249 (b-CH_2_+SO_3_), 285.04367 (c-CH_2_+SO_3_), 315.05338 (d-CH_2_+SO_3_), and 421.09387 (e-2CH_2_+SO_3_) suggested that sulfation occurred on the guaiacol moiety. The product ions at *m*/*z* 137.06032 (a-CH_2_), 205.08562 (c-CH_2_), 235.09645 (d-CH_2_), and 341.13934 (e-2CH_2_) were consistent with the fragment ions of M3 (Figure S1). M3-S1 and M3-S2 were assigned as sulfate conjugates of M3 based on a comparison of the retention time and MS/MS fragmentation with pinoresinol sulfate generated by the incubation of pinoresinol with human liver S9 fractions in the presence of PAPS (Figure S2D).

### 3.3. Contributions of CYP Enzymes to the Phase I Metabolism of Eudesmin

**Figure 4:**
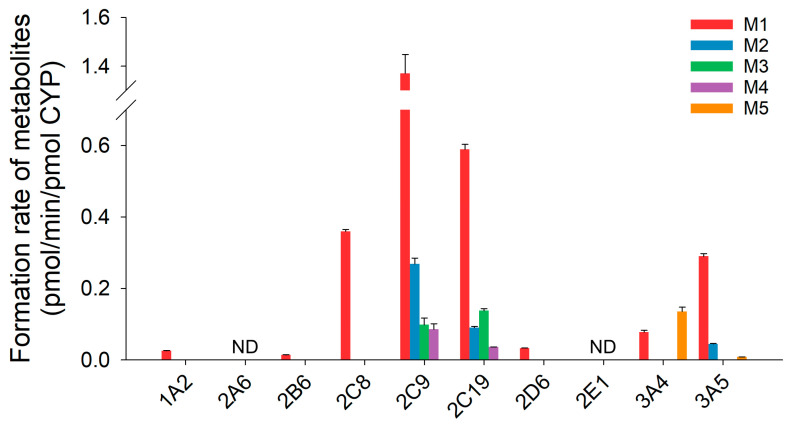
Formation rates of eudesmin phase I metabolites (M1−M5) after incubation with 5 µM eudesmin and human cDNA-expressed CYP isozymes in the presence of NADPH at 37 °C for 30 min. Each data point represents the mean ± SD (*n*  =  3). ND: not detected (lower limit of quantification: 0.002 pmol/min/pmol CYP).

To identify the CYP isozymes involved in phase I metabolism of eudesmin, cDNA-expressed human CYP isozymes were used (Figure 4). For M1 formation, CYP2C9 showed the highest activity, followed by CYP2C19, CYP2C8, and CYP3A5. M2 was formed by CYP2C9, CYP2C19, and CYP3A5. M3 and M4 were formed by CYP2C9 and CYP2C19. In contrast, M5 was primarily formed by CYP3A4.

**Figure 5:**
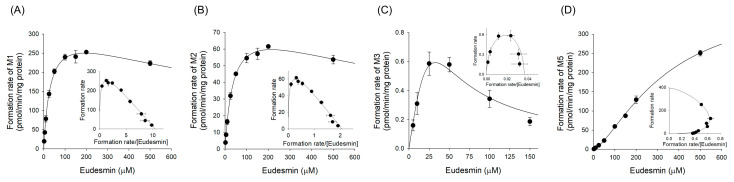
Concentration-dependent formation rates of (**A**) M1, (**B**) M2, (**C**) M3, and (**D**) M5 in HLM over the concentration range of 2–500 μM eudesmin. The insets are Eadie–Hofstee plots. Each data point represents the mean ± SD (*n*  =  3).

Subsequently, enzyme kinetic analyses were performed in HLM to characterize the formation of individual metabolites (Figure 5). Eadie–Hofstee plots showed that the formation of M1, M2, and M3 followed the substrate inhibition kinetics, whereas M5 exhibited Hill kinetics. Table 3 lists the corresponding kinetic parameters. Based on the intrinsic clearance (*CL*_int_) values, M1 showed the highest clearance (10.4 μL/min/mg protein), followed by M2 (2.1 μL/min/mg protein) and M5 (1.2 μL/min/mg protein). In contrast, M3 showed the lowest intrinsic clearance (0.04 μL/min/mg protein) among the metabolites.

The CYP isozymes contributing to eudesmin metabolism were identified by determining the enzyme kinetic parameters in human cDNA-expressed CYP2C8, CYP2C9, CYP2C19, CYP3A4, and CYP3A5 (Table 3). For M1 formation, CYP2C9 and CYP3A5 contributed to eudesmin metabolism, whereas CYP2C8, CYP2C19, and CYP3A4 showed minor contributions. M2 formation was primarily mediated by CYP2C9, with minor contributions from CYP2C19, CYP3A4, and CYP3A5. For M3 formation, CYP2C9 and CYP2C19 contributed to the metabolism. A similar pattern was observed for M4 formation, with contributions from CYP2C9 and CYP2C19. In contrast, M5 formation was mediated by CYP3A4 and CYP3A5.

**Figure 6:**
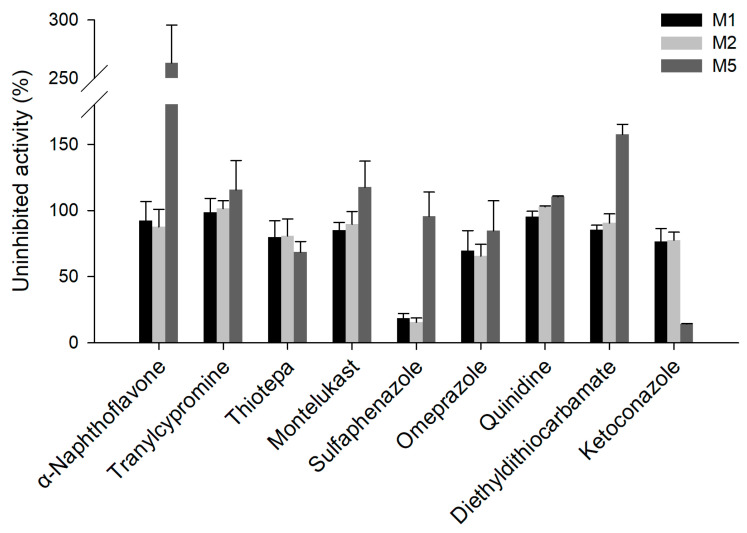
Chemical inhibition of CYP enzymes involved in the formation of M1, M2, and M5 from eudesmin in HLM using the selective inhibitors for CYP1A2 (α-naphthoflavone), CYP2A6 (tranylcypromine), CYP2B6 (thiotepa), CYP2C8 (montelukast), CYP2C9 (sulfaphenazole), CYP2C19 (omeprazole), CYP2D6 (quinidine), CYP2E1 (diethyldithiocarbamate), and CYP3A4 (ketoconazole). Each data point represents the mean ± SD (*n*  =  3).

Chemical inhibition experiments were conducted in NADPH-supplemented HLM to clarify the CYP enzymes involved in the phase I metabolism of eudesmin (Figure 6). Treatment with sulfaphenazole, a selective CYP2C9 inhibitor, resulted in remaining activities of 18.7% and 15.0% for M1 and M2 formation, respectively. Inhibition with omeprazole, a selective CYP2C19 inhibitor, led to remaining activities of 69.5% and 65.5% for M1 and M2 formation, respectively. Treatment with the CYP2C8 inhibitor resulted in 85% residual activity for M1 formation. Furthermore, treatment with ketoconazole, a potent CYP3A4 inhibitor, resulted in 76.4% and 77.6% residual activities for M1 and M2 formation, respectively, and 14.2% for M5 formation. In contrast, the formation of the CYP3A-mediated metabolite M5 was enhanced by more than 250% in the presence of α-naphthoflavone. Overall, the inhibition profiles were in agreement with the enzyme kinetic data regarding CYP isoform involvement in eudesmin metabolism. The formation of M3 and M4 was limited under the tested substrate concentration, and therefore, these metabolites were not quantified in chemical inhibition experiments.

### 3.4. UGT and SULT Enzyme Screening for Phase II Metabolism of Eudesmin

**Figure 7:**
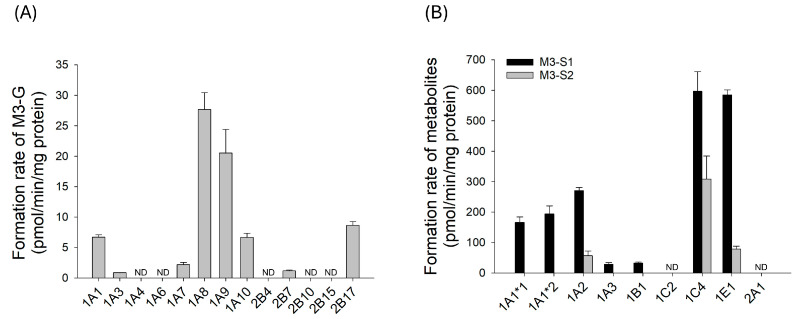
Formation rates of (**A**) M3-G (pinoresinol glucuronide) and (**B**) M3-S1 (pinoresinol sulfate) and M3-S2 (pinoresinol disulfate) from 5 µM pinoresinol in human cDNA-expressed UGT and SULT isoforms, respectively. The data represents mean ± SD (*n*  =  3). ND: not detected (lower limit of quantification: 0.2 pmol).

The phase II metabolism of eudesmin was examined through UGT and SULT enzyme screening using the available authentic standard M3 (pinoresinol) (Figure 7). Glucuronide formation of M3 (M3-G) was mediated primarily by UGT1A8 and UGT1A9. In addition, UGT1A1, UGT1A3, UGT1A7, UGT1A10, UGT2B7, and UGT2B17 also contributed to M3-G formation. Sulfate conjugation of M3 resulted in the formation of two sulfate metabolites, M3-S1 and M3-S2. M3-S1 was formed by SULT1A1*1, SULT1A1*2, SULT1A2, SULT1C4, and SULT1E1. M3-S2 formation was observed for SULT1A2, SULT1C4, and SULT1E1. These results suggest that multiple UGT and SULT isoforms are involved in the phase II conjugation of M3.

**Figure 8:**
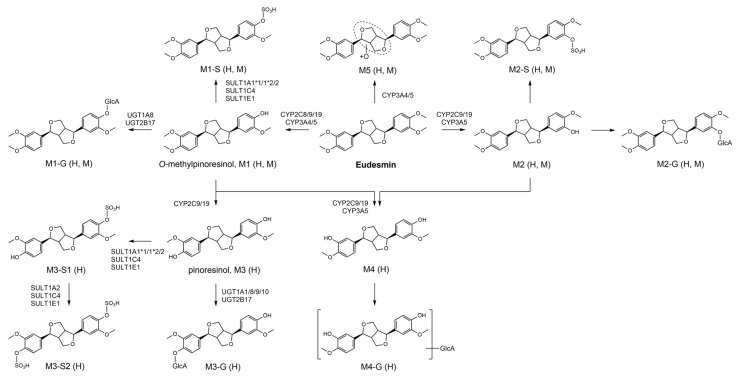
Metabolic pathways of eudesmin in human (H) and mouse (M) hepatocytes.

Based on the collective findings from metabolite identification and enzyme contribution analysis involving CYP, UGT, and SULT enzymes, the in vitro metabolic pathways of eudesmin in human and mouse hepatocytes were proposed (Figure 8). For M1-S and M1-G, the phase II metabolic enzymes responsible for the sulfation and glucuronidation of M1 (*O*-methylpinoresinol) have been previously reported. Therefore, these pathways were incorporated based on the literature [18].

## 4. Discussion

The comprehensive characterization of eudesmin metabolism in this study was facilitated by the application of IIMN. IIMN enabled the identification of minor and unexpected metabolites sharing characteristic fragmentation patterns with eudesmin by clustering metabolites based on MS/MS spectral similarity and integrating ion identity information. This strategy provided a comprehensive overview of eudesmin biotransformation pathways (Figure 1).

Eudesmin was metabolized into 13 metabolites in human hepatocytes, including five phase I metabolites (M1–M5) and eight phase II conjugates (Table 2 and Figure 8). Phase I metabolism was dominated by *O*-demethylation of the 3,4-dimethoxyphenyl moieties, yielding mono- (M1 and M2) and di-*O*-demethylated metabolites (M3 and M4) that introduce guaiacol moieties, along with a hydroxylated metabolite (M5). These findings suggest that the 3,4-dimethoxyphenyl moiety represents a metabolic hotspot for CYP-mediated metabolism, consistent with the reported metabolic profiles of structurally related tetrahydrofurofuranoid lignans such as magnolin, kobusin, fargesin, and aschantin [14,16,17,18].

Enzyme phenotyping, kinetic analyses, and chemical inhibition experiments identified CYP2C9 as the major contributor to *O*-demethylation, with additional contributions from CYP2C19, CYP2C8, and CYP3A isoforms depending on the metabolite (Table 3, Figure 4 and Figure 6). Among these, M1 formation was the predominant pathway, showing the highest intrinsic clearance in HLM (Table 3 and Figure 5). These results suggest that eudesmin metabolism involves multiple CYP enzymes, a feature commonly observed for tetrahydrofurofuranoid lignans. Across tetrahydrofurofuranoid lignans, *O*-demethylation is frequently mediated by CYP2C9, often with concurrent contributions from CYP2C8, 2C19 and CYP3A enzymes [14,16,17,18]. In contrast, hydroxylation on the tetrahydrofuran ring appeared to involve CYP3A isoforms, consistent with previous reports on structurally related tetrahydrofurofuranoid lignans such as aschantin and kobusin (Table 3, Figure 4 and Figure 6) [16,18]. Interestingly, treatment with α-naphthoflavone enhanced M5 formation (Figure 7). Although α-naphthoflavone is a widely recognized chemical inhibitor of CYP1A2, it has also been reported to act as a potent allosteric activator of CYP3A4, which is consistent with the observed increase in M5 formation [30].

A significant finding of this study is the identification of M3 as pinoresinol, a bioactive lignan reported to exhibit antioxidant, anti-inflammatory, and hypoglycemic activities [31,32,33]. The extensive formation of M3 in human hepatocytes suggests that the in vivo therapeutic effects of eudesmin might not be solely attributed to the parent compound itself, but to its active metabolites. Hence, eudesmin may function as a prodrug or exhibit synergistic effects with its metabolites, a factor that must be considered when interpreting its clinical efficacy.

Di-*O*-demethylated metabolites (M3 and M4) and their conjugates (M3-G, M4-G, M3-S1, and M3-S2) were detected in human hepatocytes but not in mouse hepatocytes, indicating the reduced formation of sequentially *O*-demethylated metabolites in mice (Table 2 and Figure 2). Similar species-specific metabolic patterns have been reported for structurally related lignan, such as magnolin, in which di-*O*-demethylated metabolites were detected in human but not rat hepatocytes [15]. These interspecies differences may arise from differences in the expression and activity of CYP2C isoforms between rodents and humans, mandating caution when extrapolating rodent data for eudesmin and structurally related tetrahydrofurofuranoid lignans to humans [34].

Phase II metabolism of eudesmin comprised glucuronidation and sulfation of all *O*-demethylated metabolites, and the enzymes responsible for the conjugation of M1 and M3 were characterized (Figure 7 and Figure 8). UGT screening suggested that glucuronidation was mediated by multiple UGT isoforms. The involvement of the intestinal (UGT1A8 and UGT1A10) and hepatic (UGT1A1, UGT1A9, and UGT2B17) UGT isoforms suggests that glucuronidation of phase I metabolites of eudesmin may occur during intestinal and hepatic metabolism, potentially limiting the systemic exposure of unconjugated metabolites following oral administration [35,36,37]. Sulfation also contributed to the phase II metabolism of eudesmin and was catalyzed by multiple SULT enzymes, including SULT1A1, SULT1A2, SULT1C4, and SULT1E1. Similarly, sulfation of guaiacol-containing tetrahydrofurofuranoid lignans, including sylvatesmin and piperitol, has been reported to involve a highly similar set of SULT isoforms [17,18].

The estimated hepatic extraction ratio for eudesmin ranged from 0.59 to 0.61, indicating that it is an intermediate extraction drug. This suggests that eudesmin is susceptible to a moderate-to-high hepatic first-pass effect, which may limit its systemic bioavailability upon oral administration. From a clinical perspective, drugs with intermediate extraction ratios are sensitive to fluctuations in hepatic blood flow and metabolic enzyme activity, potentially leading to inter-individual pharmacokinetic variability [38]. While previous studies on eudesmin have focused on its perpetrator potential, the present study provides new information on its potential to act as a victim of DDIs by identifying the major metabolic pathways and quantitatively characterizing the CYP isoforms responsible for its clearance [12,13]. Our findings reveal that M1 formation is the predominant metabolic pathway, mediated mainly by CYP2C9 (55.7%) and CYP3A5 (38.8%) (Table 3). Furthermore, M2 formation was almost exclusively driven by CYP2C9 (96.0%) (Table 3). These results demonstrate that eudesmin clearance is highly dependent on CYP2C9. Therefore, co-administration with potent CYP2C9 inhibitors, such as fluconazole or amiodarone, may substantially increase systemic exposure to eudesmin [39]. Such interactions warrant clinical caution, particularly in patients with reduced CYP2C9 activity due to genetic polymorphisms or those under polypharmacy regimens [40].

In conclusion, eudesmin undergoes extensive biotransformation in human hepatocytes, yielding five phase I and eight phase II metabolites. The metabolic pathway is characterized by CYP2C9-mediated *O*-demethylation and CYP3A4-mediated hydroxylation, followed by broad-spectrum conjugation by UGT and SULT enzymes. The identification of pinoresinol as a major human metabolite and the observation of significant interspecies differences provide critical insights into the pharmacokinetics of eudesmin. These findings establish a definitive metabolic framework for eudesmin, supporting its further pharmaceutical development and clinical evaluation.

## Figures and Tables

**Table 1:** Metabolic stability parameters of eudesmin in human and mouse hepatocytes. Data are presented as mean ± SD (*n  *=  3).

Parameters	Human	Mouse
*t*_1/2_ (min)	181.0 ± 25.3	132.9 ± 2.4
*CL*_int_ (mL/min/kg)	27.7 ± 3.6	154.0 ± 2.9
*CL*_hep_ (mL/min/kg)	11.8 ± 0.7	56.8 ± 0.4
Hepatic extraction ratio	0.57 ± 0.03	0.63 ± 0.01

**Table 2:** Retention time (t_R_), elemental composition, molecular ion ([M+H]^+^ or [M+NH_4_]^+^), product ion, and biotransformation of eudesmin and its metabolites in human (H) and mouse (M) hepatocytes.

Name	t_R_ (min)	Elemental Composition	*m*/*z* [M+NH_4_]^+^	Mass Error (ppm)	Product Ions (*m*/*z*)	Biotransformation	Species	
							H	M
Eudesmin	43.22	C_22_H_26_O_6_	404.20651	−0.64	151.07539, 189.09100, 219.10158, 249.11201, 369.16962	-	○	○
M1	36.87	C_21_H_24_O_6_	390.19128	0.41	137.05957, 151.07521, 175.07524, 189.09074, 205.08592, 219.10138, 235.09628, 249.11179, 355.15372	*O*-demethylation	○	○
M2	39.4	C_21_H_24_O_6_	390.19107	−0.13	137.06009, 151.07590, 175.07573, 189.09126, 205.08585, 219.10115, 235.09683, 249.11234, 355.15302	*O*-demethylation	○	○
M3	25.76	C_20_H_22_O_6_	376.17499	−1.28	137.05969, 175.07527, 205.08578, 235.09630, 341.13824	di-*O*-demethylation	○	ND
M4	30.19	C_20_H_22_O_6_	376.17520	−0.72	137.05963, 175.07526, 205.08604, 235.09630, 341.13779	di-*O*-demethylation	○	ND
M5	35.55	C_22_H_26_O_7_	403.17538 *	0.62	151.07532, 189.09097, 217.08586, 219.10172, 247.09645, 265.10626, 385.16425	hydroxylation	○	○
M1-G	13.56	C_27_H_32_O_12_	566.22369	0.85	137.05952, 151.07536, 175.07541, 189.09113, 205.08612, 219.10159, 235.09647, 249.11214, 355.15408	*O*-demethylation & glucuronidation	○	○
M2-G	20.25	C_27_H_32_O_12_	566.22400	1.40	137.05969, 151.07545, 175.07542, 189.09100, 205.08607, 219.10155, 235.09637, 249.11203, 355.15399	*O*-demethylation & glucuronidation	○	○
M3-G	5.43	C_26_H_30_O_12_	552.20862	1.92	137.05975, 175.07547, 205.08609, 235.09648, 341.13846	di-*O*-demethylation & glucuronidation	○	ND
M4-G	11.53	C_26_H_30_O_12_	552.20807	0.92	175.07616, 205.08626, 235.09628, 341.13971	di-*O*-demethylation & glucuronidation	○	ND
M1-S	20.02	C_21_H_24_O_9_S	470.14838	0.94	137.05954, 151.07553, 175.07542, 217.01657, 235.09653, 249.11153, 255.03215, 285.04260, 315.05359, 355.15414, 435.11111	*O*-demethylation & sulfation	○	○
M2-S	25.41	C_21_H_24_O_9_S	470.14764	−0.64	217.01633, 235.09778, 249.11172, 285.04147, 315.05350, 355.15515, 435.11029	*O*-demethylation & sulfation	○	○
M3-S1	10.74	C_20_H_22_O_9_S	456.13239	0.22	137.05975, 175.07561, 205.08652, 217.01608, 235.09636, 255.03220, 285.04297, 315.05316, 341.13803, 421.09561	di-*O*-demethylation & sulfation	○	ND
M3-S2	2.51	C_20_H_22_O_12_S_2_	536.08972	1.16	137.06032, 205.08562, 217.01642, 235.09645, 255.03249, 285.04367, 315.05338, 341.13934, 421.09387, 456.13095	di-*O*-demethylation & disulfation	○	ND

○, detected; ND, not detected; *, [M+H]^+^.

**Table 3:** Enzyme kinetic parameters for the metabolism of eudesmin in HLM and recombinant CYP enzymes.

Parameters	CYP2C8	CYP2C9	CYP2C19	CYP3A4	CYP3A5	HLM
M1						
*K*_m_ (μM)	16.6	18.9	21.2	123.9	276.4	32.0
*V*_max_	0.8	3.3	1.0	2.9	15.8	333.9
*K*_i_ (μM)	-	911.0	1616.4	-	-	1154.8
*CL*_int_	0.05	0.2	0.05	0.02	0.06	10.4
*n*	-	-	-	1.6	-	-
Contribution (%)	3.8	55.7	0.3	5.2	38.8	-
M2						
*K*_m_ (μM)	-	31.2	46.4	154.0	70.6	39.6
*V*_max_	-	3.6	0.5	0.6	0.6	81.9
*K*_i_ (μM)	-	197.9	96.7	-	1297.7	1141.7
*CL*_int_	-	0.1	0.01	0.004	0.008	2.1
*n*	-	-	-	1.5	-	1.4
Contribution (%)	-	96.0	0.2	1.6	2.2	-
M3						
*K*_m_ (μM)	-	6.4	114.0	-	-	25,404.9
*V*_max_	-	0.02	0.9	-	-	925.0
*K*_i_ (μM)	-	56.1	3.8	-	-	0.04
*CL*_int_	-	0.004	0.008	-	-	0.04
Contribution (%)	-	63.3	36.7	-	-	-
M4						
*K*_m_ (μM)	-	6.9	1899.9	-	-	-
*V*_max_	-	0.02	3.2	-	-	-
*K*_i_ (μM)	-	70.3	0.3	-	-	-
*CL*_int_	-	0.003	0.002	-	-	-
*n*	-	-	-	-	-	-
Contribution (%)	-	30.4	69.6	-	-	-
M5						
*K*_m_ (μM)	-	-	-	139.0	164.9	339.5
*V*_max_	-	-	-	10.3	2.2	394.7
*CL*_int_	-	-	-	0.07	0.01	1.2
*n*	-	-	-	1.4	1.3	1.4
Contribution (%)	-	-	-	77.3	22.7	-

*V*_max_: pmol/min/pmol CYP for CYP isozymes and pmol/min/mg protein for HLM; *CL*_int_: μL/min/pmol CYP isozymes and μL/min/mg protein for HLM; *n*: Hill coefficient; -: not calculated.

## Data Availability

The original contributions presented in this study are included in the article. Further inquiries can be directed to the corresponding author.

## Supplementary Materials

The following supporting information can be downloaded at https://www.mdpi.com/article/10.3390/pharmaceutics18040432/s1; Figure S1: MS/MS spectra of M1-G, M2-G, M3-G, M4-G, M1-S, M2-S, M3-S1, and M3-S2; Figure S2: Extracted ion chromatograms of metabolites formed from M1 (*O*-methylpinoresinol) and M3 (pinoresinol) following incubation with human liver S9 fractions in the presence or absence of UDPGA or PAPS; Figure S3: Extracted ion chromatograms of eudesmin metabolites after enzymatic hydrolysis of human hepatocytes incubation mixtures. Table S1: Metabolic stability parameters of honokiol as a positive control in human and mouse hepatocytes. Data are presented as mean ± SD (*n* = 3).
