# Supplementary material for: Comprehensive In Vitro Metabolic Characterization of Eudesmin in Human and Mouse Hepatocytes
Source: Pharmaceutics. 2026 Mar 31;18(4):432. doi: 10.3390/pharmaceutics18040432 (PMC13119212; doi:10.3390/pharmaceutics18040432)
Supplement: Supplementary file 1 [file pharmaceutics-18-00432-s001.zip › pharmaceutics-4199383-supplementary.pdf]

Supplementary Materials

Comprehensive In Vitro Metabolic Characterization of Eudesmin in Human and Mouse Hepatocytes

Min Seo Lee , Ju-Hyun Kim , Im-Sook Song, Yong-Yeon Cho, Joo Young Lee, and Hye Suk Lee

Table S1. Metabolic stability parameters of honokiol as a positive control in human and mouse hepatocytes. Data are presented as mean ± SD ($n = 3$).

Parameters	Human	Mouse
$t_{1/2}$ (min)	14.8 ± 0.8	15.1 ± 2.2
CL_{int} (mL/min/kg)	336.0 ± 17.9	1370.1 ± 200.5
CL_{hep} (mL/min/kg)	19.5 ± 0.1	84.4 ± 0.8
Hepatic extraction ratio	0.94 ± 0.01	0.94 ± 0.01

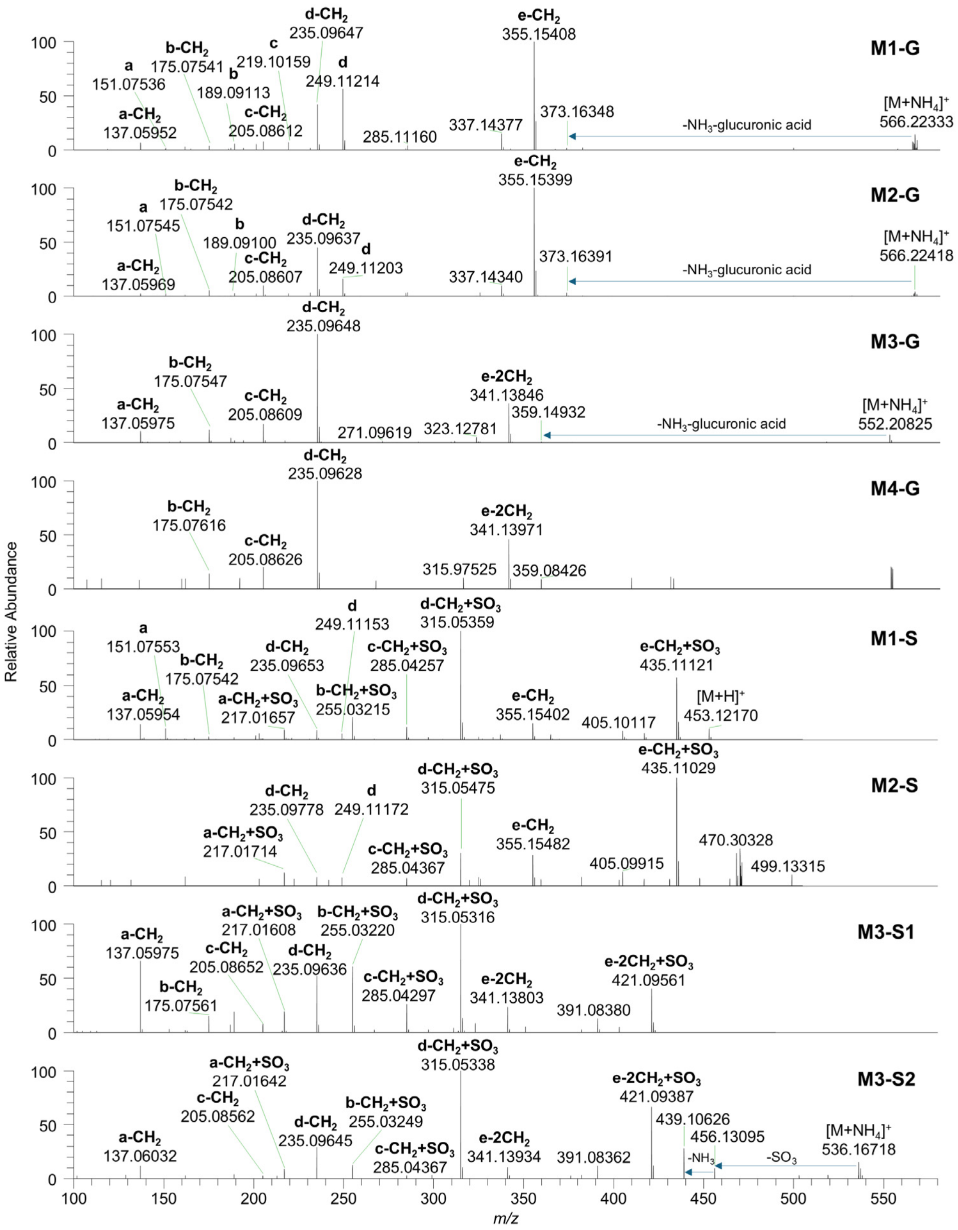

Figure S1. MS/MS spectra of M1-G, M2-G, M3-G, M4-G, M1-S, M2-S, M3-S1, and M3-S2.

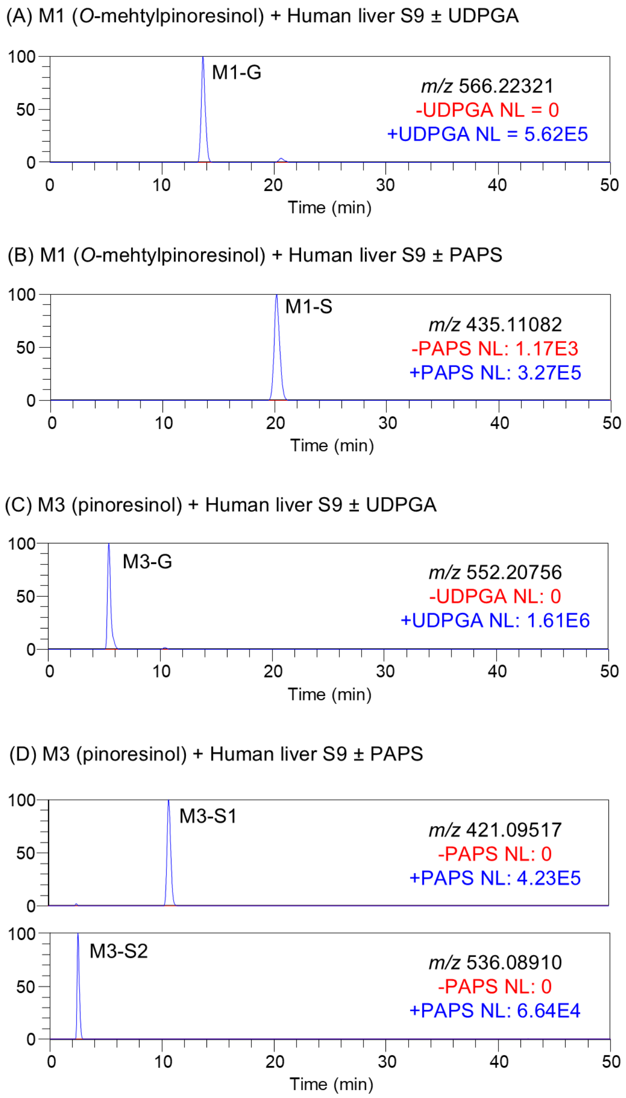

Figure S2. Extracted ion chromatograms of metabolites formed from M1 (*O*-methylpinoresinol) and M3 (pinoresinol) following incubation with human liver S9 fractions in the presence or absence of UDPGA or PAPS. Red lines indicate incubations without cofactors, and blue lines indicate incubations with UDPGA or PAPS.

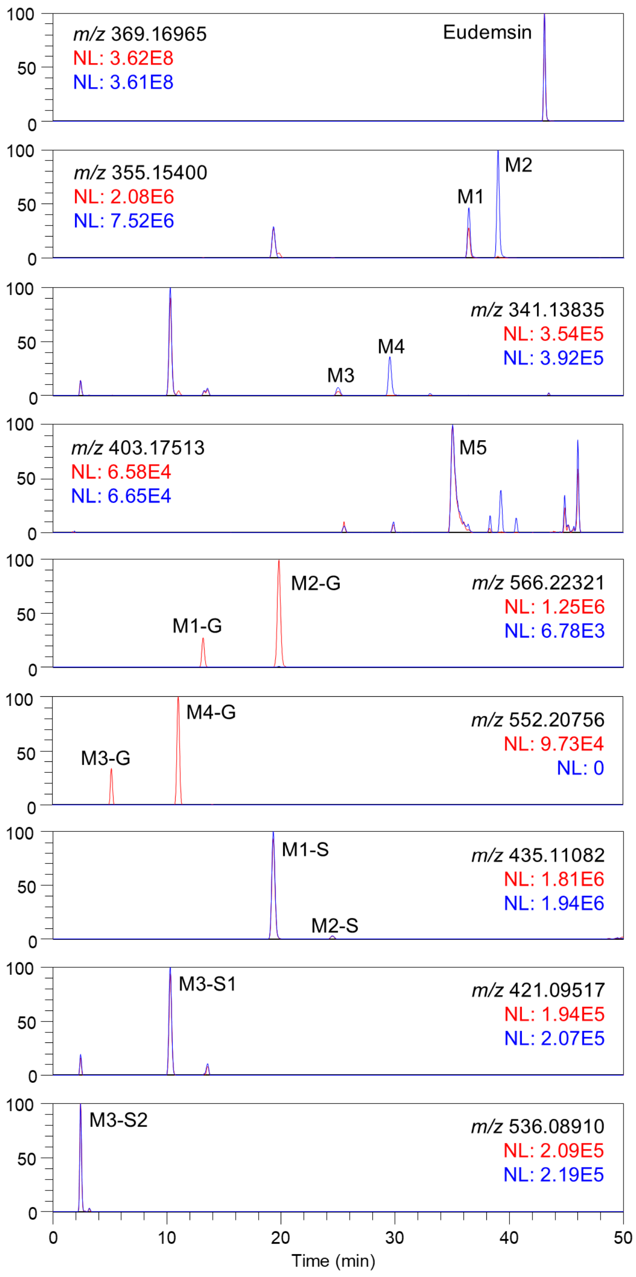

Figure S3. Extracted ion chromatograms of eudesmin metabolites after enzymatic hydrolysis of human hepatocytes incubation mixtures. The red line represents samples without β-glucuronidase, and the blue line represents samples treated with β-glucuronidase.
